# 可解释性深度学习算法在肺占位性病变良恶性诊断及肺癌病理亚型分类中的运用

**DOI:** 10.3779/j.issn.1009-3419.2025.102.36

**Published:** 2025-10-20

**Authors:** Haoran LI, Yuanyuan WANG, Yang WANG, Huihui HE, Junya LI, Yanning SU, Fanrui KONG, Xiangli LIU, Liuhui CHENG, Ya LI

**Affiliations:** ^1^450000 郑州，河南中医药大学第一临床医学院（李浩然，李俊雅，苏艳宁，李亚）; ^1^First Clinical Medical College, Henan University of Chinese Medicine; ^2^河南中医药大学第一附属医院国家区域中医（肺病）诊疗中心（李浩然，王元元，王洋，李俊雅，苏艳宁，李亚）; ^2^National Regional Diagnosis and Treatment Center for Traditional Chinese Medicine (Pulmonary Diseases), The First Affiliated Hospital of Henan University of Chinese Medicine; ^3^河南中医药大学第一附属医院实验中心，中药药理（呼吸）实验室，河南省呼吸病防治中医药重点实验室（何慧慧，李亚）; ^3^Experimental Center, The First Affiliated Hospital of Henan University of Chinese Medicine; Pharmacology of Chinese Materia Medica (Respiration) Laboratory; Henan Provincial Key Laboratory of Chinese Medicine for Respiratory Disease Prevention and Treatment; ^4^河南中医药大学第二临床医学院（孔繁睿）; ^4^Second Clinical Medical College, Henan University of Chinese Medicine; ^5^河南中医药大学第一附属医院病理科（刘香丽）; ^5^Department of Pathology, The First Affiliated Hospital of Henan University of Chinese Medicine; ^6^河南中医药大学第一附属医院放射科（程留慧）; ^6^Department of Radiology, The First Affiliated Hospital of Henan University of Chinese Medicine; ^7^河南中医药大学呼吸疾病中医药防治省部共建协同创新中心（李亚）; ^7^Provincial and Ministerial Collaborative Innovation Center for the Prevention and Treatment of Respiratory Diseases with Chinese Medicine, Henan University of Chinese Medicine, Zhengzhou 450000, China

**Keywords:** 肺肿瘤, 肺占位性病变, 机器学习, 特征诠释, 良恶性诊断, 深度学习, Lung neoplasms, Pulmonary space-occupying lesion, Machine learning, Feature interpretation, Benign-malignant diagnosis, Deep learning

## Abstract

**背景与目的:**

肺占位性病变的良恶性鉴别与肺癌病理亚型分类是临床决策的关键，但传统方法存在多源临床数据利用不足及深度学习模型可解释性差的问题。本研究基于针对表格化数据设计的Transformer（Tab-Transformer）与残差多层感知器（Residual Multi-Layer Perceptron, ResMLP）的混合架构（TT-ResMLP），探讨可解释性深度学习算法在肺占位性病变良恶性诊断及肺癌病理亚型分类中的性能。

**方法:**

收集345例经病理证实的肺占位性病变患者的影像学特征、病史资料及实验室检查等数据，按8:2随机分为训练集和测试集。采用*Spearman*检验与最小绝对收缩和选择算子（Least Absolute Shrinkage and Selection Operator, *LASSO*）筛选稳定特征，使用合成少数类过采样技术（Synthetic Minority Over-sampling Technique, SMOTE）平衡样本，采用10折交叉验证提高模型泛化能力，选用Tab-Transformer算法、ResMLP算法、TT-ResMLP构建模型，通过受试者工作特征（receiver operating characteristic, ROC）曲线、曲线下面积（area under the curve, AUC）、准确率、特异性、敏感性和微平均ROC（micro-averaged ROC, micro-ROC）曲线评估模型性能，并基于最优模型进行SHAP（SHapley Additive exPlanations）特征分析。

**结果:**

良恶性诊断模型中，3种模型均表现良好，其中Tab-Transformer在测试集表现最优，TT-ResMLP和ResMLP次之；SHAP分析显示，表现最优的Tab-Transformer模型特征重要性依次是年龄、胸膜凹陷征、凝血酶时间、平均密度、磨玻璃样改变等，其中胸膜凹陷征有较高的恶性诊断贡献，且随年龄增长、凝血酶时间缩短，其贡献度进一步增强。在肺癌亚型分类任务中，3种模型均表现出优异性能，其中TT-ResMLP综合表现最优。SHAP分析进一步揭示，肺部影像报告和数据系统评分（Lung Imaging Reporting and Data System, Lung-RADS）在3种病理亚型中均具较高重要性；男性与鳞癌预测呈正相关；神经元特异性烯醇化酶（neuron-specific enolase, NSE）在小细胞癌预测中起重要作用。在腺癌中，诊断概率与Lung-RADS分级呈正相关，且在低凝血酶原时间值时更显著；而在鳞癌与小细胞癌亚组中呈负相关，但性别和NSE水平可增强其风险预测的贡献。特征决策边界分析显示，Lung-RADS分级在腺癌识别中具有较高的区分能力，而NSE在小细胞癌识别中展现出更强的区分能力。

**结论:**

TT-ResMLP混合架构能达到肺占位性病变的良恶性诊断及肺癌病理亚型分类的目的，模型具备良好的可解释性，有助于识别关键预测特征并揭示其交互机制，为深入理解肺癌生物学行为及临床辅助决策提供了有效工具。

## Abstract (en)

**Background and objective:**

The discrimination between benign and malignant pulmonary space-occupying lesions and the classification of pathological subtypes of lung cancer are critical for clinical decision-making. However, conventional methods often suffer from insufficient utilization of multi-source clinical data and poor interpretability of deep learning models. This study investigates the performance of interpretable deep learning algorithms in diagnosing benign versus malignant pulmonary space-occupying lesions and classifying pathological subtypes of lung cancer, using a hybrid architecture based on Tab-Transformer-designed for tabular data and Residual Multi-Layer Perceptron (ResMLP), referred to as TT-ResMLP.

**Methods:**

Data including radiological characteristics, medical history, and laboratory findings from 345 patients with pathologically confirmed pulmonary space-occupying lesions were collected. The dataset was randomly split into a development set and a test set at an 8:2 ratio. Stable features were selected using the *Spearman* correlation test and the Least Absolute Shrinkage and Selection Operator (*LASSO*). The Synthetic Minority Over-sampling Technique (SMOTE) was employed to balance the samples, and 10-fold cross-validation was used to enhance model generalizability. Models were constructed using the Tab-Transformer algorithm, the ResMLP algorithm, and the TT-ResMLP hybrid. Model performance was evaluated using receiver operating characteristic (ROC) curves, the area under the curve (AUC), accuracy, specificity, sensitivity, and micro-averaged ROC (micro-ROC). SHapley Additive exPlanations (SHAP) analysis was performed based on the optimal model.

**Results:**

In the benign *vs* malignant diagnosis task, all three models performed well. The Tab-Transformer model demonstrated the best performance on the test set, followed by TT-ResMLP and ResMLP. SHAP analysis of the top-performing Tab-Transformer model revealed that the feature importance ranking was: age, pleural indentation, thrombin time, mean density, and ground-glass opacity. Pleural indentation contributed substantially to malignant diagnosis, and its contribution was further enhanced with increasing age and decreasing thrombin time. In the lung cancer subtype classification task, all three models exhibited excellent performance, with the TT-ResMLP hybrid showing the best overall performance. SHAP analysis further revealed that the Lung Imaging Reporting and Data System (Lung-RADS) category held high importance across all three pathological subtypes. Male gender was positively associated with the prediction of squamous cell carcinoma. Neuron-specific enolase (NSE) played a significant role in predicting small cell carcinoma. For adenocarcinoma, the diagnostic probability was positively correlated with the Lung-RADS category, a relationship more pronounced at lower prothrombin time (PT) values. In contrast, a negative correlation was observed in the squamous cell carcinoma and small cell carcinoma subgroups, although gender and NSE levels could enhance their contributory risk prediction. Analysis of feature decision boundaries indicated that the Lung-RADS grade possessed high discriminative power for identifying adenocarcinoma, whereas NSE demonstrated stronger discriminative ability for identifying small cell carcinoma.

**Conclusion:**

The TT-ResMLP hybrid architecture is effective for diagnosing the benign or malignant nature of pulmonary space-occupying lesions and classifying pathological subtypes of lung cancer. The model possesses good interpretability, aiding in the identification of key predictive features and unravelling their interactive mechanisms, thereby providing an effective tool for a deeper understanding of lung cancer biology and clinical decision support.

全球肺癌5年生存率低于36%，尽管I期患者生存率可达77%-92%，但早期诊断仍是重大难题^[1]^。在我国，肺癌患者早期诊断比例低于20%。因此，实现早诊、早治是提升患者生存率和改善预后的关键^[2]^。目前，肺癌诊断仍局限于影像学、肿瘤标志物筛查、生化指标等单一模态特征分析，如影像学特征在肺癌诊断中具有重要价值，毛刺、分叶、血管征、胸膜牵拉及凹陷征提示恶性倾向^[3,4]^，高脂肪占比^[5]^、爆米花样改变^[6]^则提示良性可能；且不同放射科医生对影像学特征的判断，存在观察者间差异^[7]^，不能很好地实现肺癌早期诊断。

以深度学习为代表的机器学习算法能够从复杂的医学影像数据中提取高维特征，客观、精准地获取肺癌生物学特征，辅助临床医生进行更准确的诊断和分类^[8]^。但现有深度学习模型决策过程缺乏透明性，难以直观展示特征对预测结果间的交互关系，降低了临床医生对模型结果的信任^[9]^。SHAP（SHapley Additive exPlanations）分析是一种基于博弈论的模型解释方法，能够清晰展示特征对模型输出的影响^[10]^。因此，本研究基于SHAP分析，实现对Tab-Transformer混合架构下模型的解释，探讨各特征在肺癌诊断和亚型分类中的性能。

## 1 资料与方法

### 1.1 诊断标准

原发性肺癌诊断标准参照《中华医学会肺癌临床诊疗指南（2022版）》^[11]^及《中国肺癌低剂量CT筛查指南（2023年版）》^[12]^：经手术、活检或细胞学病理确诊。

### 1.2 纳入标准

（1）经手术、穿刺活检、支气管镜下活检或细胞学病理确诊；（2）年龄≥18岁；（3）确诊前2周内接受计算机断层扫描（computed tomography, CT）检查。

### 1.3 排除标准

（1）图像质量欠佳；（2）病理确诊为转移性病灶；（3）合并活动性肺结核、肺纤维化、支气管扩张症、肺脓肿等呼吸系统疾病。

### 1.4 数据来源

回顾性分析2022年1月1日至2023年12月31日河南中医药大学第一附属医院呼吸科、胸外科收治的345例肺占位性病变患者，包括良性95例、腺癌169例、鳞癌57例、小细胞肺癌24例。收集临床及影像学资料，共提取39种特征变量（图1）。本研究已通过河南中医药大学第一附属医院伦理委员会审核（伦理批号：2023HL-125-01）。

**图1:**
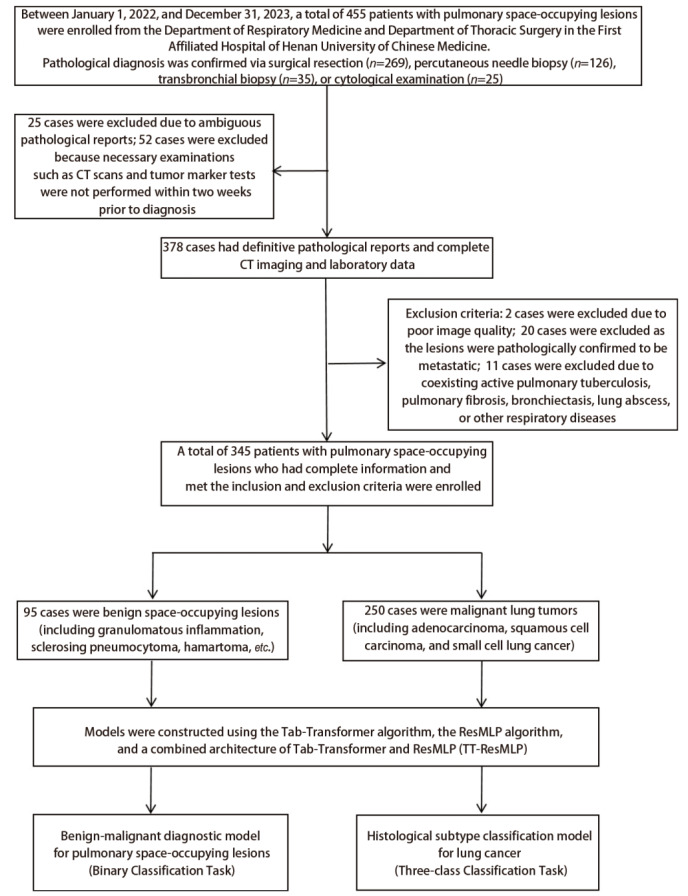
纳入/排除流程图

### 1.5 数据库的建立

#### 1.5.1 影像学评价指标获取与评估

本研究采用中国国家药品监督管理局（批准号：20232210007）和美国食品药品监督管理局（批准号：K143586）认证的医学影像量化诊断系统（陕西神州德信医学影像技术有限公司，V1.2.0），通过ResNet-CNN和Level Set算法实现肺占位性病变的检测，数据经HU校准和Z-score标准化^[13]^。最终得到肺占位性病变容积、大小、密度、质量、表面积、胸膜占比、脂肪占比、血管征、毛刺及分叶、细支气管征、钙化、胸膜牵拉及凹陷征、空泡征及肺部影像报告和数据系统评分（Lung Imaging Reporting and Data System, Lung-RADS）等影像学特征（图2）。所有特征的生成均由2名经过专业系统培训的呼吸科/影像科医师操作医学影像量化诊断系统完成。对于存在分歧的特征，由一名从事影像诊断学超过10年的主任医师进行审核与确认。

**图2:**
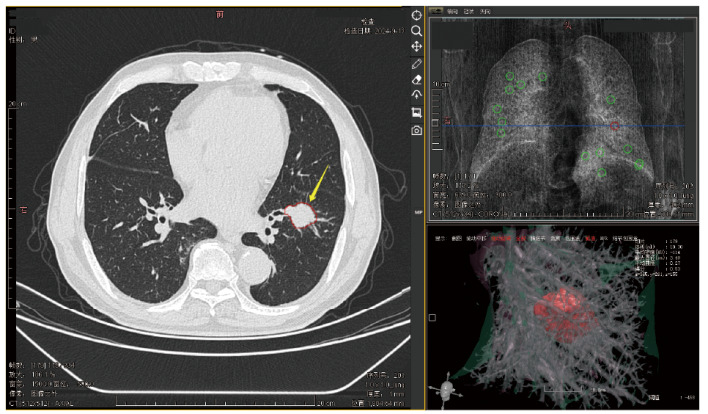
医学影像量化诊断系统工作界面及CT影像表现

#### 1.5.2 临床信息采集

从河南中医药大学第一附属医院病历系统采集患者性别、年龄、吸烟史、肿瘤史、肿瘤家族史及实验室检查指标等基线数据。

### 1.6 数据预处理

使用Python 3.12.8软件对345例肺占位性病变患者数据按8:2比例随机分为训练集与测试集，用以构建肺占位性病变良恶性诊断模型；对其中250例肺癌患者数据按8:2比例随机分为训练集与测试集，确保病理结果比例与原数据集一致，用以构建病理亚型分类模型。缺失值处理：若单个样本缺失比例<10%，定性变量用众数填充，定量变量用均数填充；缺失值>10%的特征予以剔除。

#### 1.6.1 数据标准化

对连续变量采用Z-Score标准化处理，使其转换为均值为0、标准差为1的分布，便于后续建模与分析。采用合成少数类过采样技术（Synthetic Minority Over-sampling Technique, SMOTE）平衡数据集，实现1:1的过采样比例，提升模型对少数类的识别能力^[14]^。SMOTE过采样作为预处理步骤直接集成到模型训练流程中，在训练集内生成合成样本，测试集则保持原始分布，避免数据泄露，利用t-SNE聚类与MLP分类器测试集指标（如Precision、Recall、F1-score等）验证SMOTE反映真实场景的能力。

#### 1.6.2 特征筛选

采用*Spearman*相关性检验识别并剔除数据中相关系数>0.8的高相关性特征^[15]^，降低过拟合的风险，提升模型训练的泛化能力。特征筛选采用*LASSO*回归，通过LassoCV函数在50个α参数值（10^-4^到10^0^对数空间）中进行搜索，并使用5折交叉验证确定最优的正则化参数λ，最终保留系数非零的特征用于后续建模^[16]^。

### 1.7 模型训练及评估

#### 1.7.1 模型开发环境及机器学习算法

本研究采用NVIDIA GeForce RTX 5090显卡进行模型训练与推理，基于Anaconda 2.6.4平台，使用PyTorch（2.7+CUDA 12.8版本）算法框架实现Tab-Transformer、残差MLP（Residual Multi-Layer Perceptron, ResMLP）及其混合架构（TT-ResMLP）的算法构建与优化。Tab-Transformer专为表格数据设计，在高维医学数据任务中表现优异^[17]^。ResMLP是深度学习的核心算法，通过残差连接缓解深层网络退化问题，有效学习输入与目标间的残差映射^[18]^。TT-ResMLP的混合深度学习架构，形成分阶段特征处理范式，通过Transformer编码获取高级语义特征，再输入ResMLP进行深层非线性变换，以学习更复杂的“特征-目标”映射关系^[19]^。该架构包含4层Transformer编码器，每层设8个注意力头、嵌入维度64、注意力维度32；解码器采用三层瓶颈架构（256-128-64维），每层包含批归一化、GELU激活函数和Dropout（0.3）正则化，并引入跨层残差连接以缓解梯度消失问题。训练使用Adam优化器（学习率0.001），权重衰减10^-4^，批大小为32，共训练100轮。并采用早停机制防止过拟合，所有实验均设置随机种子为42，以确保结果可复现性。

#### 1.7.2 模型训练与评估

采用10折交叉验证的方法提高模型泛化能力。训练集用于训练模型；测试集用于评估模型性能，检验过拟合的风险。采用受试者工作特征（receiver operating characteristic, ROC）曲线、曲线下面积（area under the curve, AUC）、准确率、特异性、敏感性和微平均ROC（micro-averaged ROC, micro-ROC）曲线作为评价指标。AUC值范围为0.5到1.0，数值越高表明分类效果越好；micro-ROC是一种多分类问题的模型评价指标，适合于类别分布不均的情况^[20]^。

### 1.8 SHAP值诠释

SHAP值是一种基于博弈论的模型解释方法，能够清晰展示特征对模型输出的影响，实现对Tab-Transformer、ResMLP、TT-ResMLP等模型的解释。SHAP值为负，表示该特征对模型预测结果有负向影响，降低了模型预测为特定类别的可能性；SHAP值为正，表示该特征对模型预测结果有正向影响，增加了模型预测为特定类别的可能性^[21]^。

### 1.9 统计学分析

采用Python 3.12.8软件进行数据分析与深度学习模型构建。数据处理使用pandas 2.2.3和scikit-learn 1.6.1，统计分析使用scipy 1.15.1。符合正态分布的计量资料以均数±标准差描述，不符合正态分布的计量资料以中位数（四分位数）描述，计数资料以例数或率（%）描述。对需要医师主观判别的分类特征（血管征、毛刺及分叶、细支气管征、钙化、胸膜牵拉及凹陷征、空泡征及Lung-RADS分级等）进行*Kappa*检验评估分类特征判读的一致性。

## 2 结果

### 2.1 基线数据

345例肺占位性病变患者良性病变95例，恶性病变250例（腺癌169例、鳞癌57例和小细胞肺癌24例）。患者中位年龄为64.00（55.00, 71.00）岁，男性占62.90%。影像学表现，部分实性结节占比23.77%，胸膜凹陷征占比55.07%，平均密度中位数为-112.82（-356.34, -26.60）HU，表面积中位数为18.18（5.06, 52.03）cm^3^，68.41%的患者Lung-RADS分级为4B类。实验室检查显示，纤维蛋白原（fibrinogen, FIB）中位水平为3.90（3.20, 5.11）g/L，凝血酶时间（thrombin time, TT）中位数为14.70（13.70, 15.40）s，凝血酶原时间（prothrombin time, PT）中位数为11.10（10.40, 12.00）s。肿瘤标志物分析显示病理类型特异性表达模式，腺癌组癌胚抗原（carcinoembryonic antigen, CEA）中位水平较高，小细胞肺癌组神经元特异性烯醇化酶（neuron-specific enolase, NSE）中位水平升高，而鳞癌组则在细胞角蛋白19片段（cytokeratin 19 fragment, CYFRA21-1）与鳞状细胞癌抗原（squamous cell carcinoma antigen, SCCA）上具有较高表达。需要医师主观判别的分类特征，总体Kappa值为0.984（95%CI: 0.978-0.990）。详细数据见表1。

**表1:** 不同病理类型的345例肺占位性病变患者临床基线数据

Characteristics	Total (*n*=345)	Benign (*n*=95)	Adenocarcinoma (*n*=169)	Squamous cell carcinoma (*n*=57)	Small cell lung cancer (*n*=24)
Gender					
Male	217 (62.90%)	57 (60.00%)	86 (50.89%)	53 (92.98%)	21 (87.50%)
Female	128 (37.10%)	38 (40.00%)	83 (49.11%)	4 (7.02%）	3 (12.50%)
Smoking history					
No	245 (71.01%)	70 (73.68%)	127 (75.15%)	34 (59.65%)	14 (58.33%)
Yes	100 (28.99%)	25 (26.32%)	42 (24.85%)	23 (40.35%)	10 (41.67%)
Tumor history					
No	300 (86.96%)	82 (86.32%)	148 (87.57%)	51 (89.47%)	19 (79.17%)
Yes	45 (13.04%)	13 (13.68%)	21 (12.43%)	6 (10.53%)	5 (20.83%)
Family history					
No	283 (82.03%)	81 (85.26%)	140 (82.84%)	42 (73.68%)	20 (83.33%)
Yes	62 (17.97%)	14 (14.74%)	29 (17.16%)	15 (26.32%)	4 (16.67%)
Ground-glass opacity					
No	326 (94.49%)	81 (85.26%)	165 (97.63%)	56 (98.25%)	24 (100.00%)
Yes	19 (5.51%)	14 (14.74%)	4 (2.37%)	1 (1.75%)	0 (0.00%)
Part-solid nodule					
No	263 (76.23%)	70 (73.68%)	129 (76.33%)	47 (82.46%)	17 (70.83%)
Yes	82 (23.77%)	25 (26.32%)	40 (23.67%)	10 (17.54%)	7 (29.17%)
Solid nodule					
No	101 (29.28%)	38 (40.00%)	44 (26.04%)	11 (19.30%)	7 (29.17%)
Yes	244 (70.72%)	57 (60.00%)	125 (73.96%)	46 (80.70%)	17 (70.83%)
Vessel sign					
No	85 (24.64%)	38 (40.00%)	31 (18.34%)	13 (22.81%)	3 (12.50%)
Yes	260 (75.36%)	57 (60.00%)	138 (81.66%)	44 (77.19%)	21 (87.50%)
Spiculation sign					
No	59 (17.10%)	30 (31.58%)	17 (10.06%)	10 (17.54%)	2 (8.33%)
Yes	286 (82.90%)	65 (68.42%)	152 (89.94%)	47 (82.46%)	22 (91.67%)
Bronchus sign					
No	196 (56.81%)	66 (69.47%)	90 (53.25%)	32 (56.14%)	8 (33.33%)
Yes	149 (43.19%)	29 (30.53%)	79 (46.75%)	25 (43.86%)	16 (66.67%)
Calcification					
No	205 (59.42%)	68 (71.58%)	100 (59.17%)	29 (50.88%)	8 (33.33%)
Yes	140 (40.58%)	27 (28.42%)	69 (40.83%)	28 (49.12%)	16 (66.67%)
Pleural indentation sign					
No	155 (44.93%)	59 (62.11%)	69 (40.83%)	20 (35.09%)	7 (29.17%)
Yes	190 (55.07%)	36 (37.89%)	100 (59.17%)	37 (64.91%)	17 (70.83%)
Vacuole sign					
No	205 (59.42%)	58 (61.05%）	104 (61.54%)	32 (56.14%)	11 (45.83%)
Yes	140 (40.58%)	37 (38.95%)	65 (38.46%)	25 (43.86%)	13 (54.17%)
Imaging findings					
Peripheral	238 (68.99%)	79 (83.16%)	114 (67.46%)	34 (59.65%)	13 (54.17%)
Central	107 (31.01%)	16 (16.84%)	55 (32.54%)	23 (40.35%)	11 (45.83%)
Lung-RADS category					
Category 2	31 (8.99%)	19 (20.00%)	5 (2.96%)	6 (10.53%)	1 (4.17%)
Category 3	34 (9.86%)	19 (20.00%)	8 (4.73%)	6 (10.53%)	1 (4.17%)
Category 4A	44 (12.75%)	14 (14.74%)	23 (13.61%)	7 (12.28%)	0 (0.00%)
Category 4B	236 (68.41%)	43 (45.26%)	133 (78.70%)	38 (66.67%)	22 (91.67%)
Age (yr)	64.00 (55.00, 71.00)	59.00 (52.00, 67.00)	64.50 (56.25, 72.00)	67.00 (62.75, 73.00)	65.00 (54.75, 71.00)
Volume (cm³)	4.89 (0.79, 19.82)	1.01 (0.24, 9.75)	6.17 (1.58, 18.26)	12.75 (1.26, 37.86)	18.55 (4.73, 55.35)
Maximum diameter (cm)	2.85 (1.49, 4.80)	1.61 (1.02, 3.57)	3.21 (1.92, 4.72)	4.22 (1.87, 5.51)	4.37 (3.00, 6.33)
Mean diameter (cm)	2.01 (1.14, 3.34)	1.24 (0.80, 2.40)	2.21 (1.40, 3.27)	2.83 (1.24, 3.94)	3.17 (2.32, 4.46)
Mean density (HU)	-112.82 (-356.34, -26.60)	-228.18 (-529.07, -98.64)	-96.50 (-341.65, -32.07)	-52.99 (-239.70, -11.15)	-33.14 (-146.96, -5.48)
Non-solid component percentage	0.03 (0.01, 0.12)	0.04 (0.01, 0.25)	0.03 (0.01, 0.11)	0.02 (0.00, 0.07)	0.02 (0.01, 0.05)
Mass (g)	3.41 (0.53, 16.40)	0.70 (0.12, 7.78)	4.61 (0.81, 15.42)	9.87 (0.80, 31.74)	16.20 (3.25, 42.41)
Surface area (cm²)	18.18 (5.06, 52.03)	5.85 (2.07, 30.83)	21.45 (7.92, 49.32)	40.14 (7.53, 83.83)	46.67 (17.77, 119.830)
Pleural contact area (cm²)	1.37 (0.33, 4.00)	0.44 (0.15, 2.56)	1.48 (0.60, 3.71)	2.34 (0.43, 7.11)	3.15 (1.55, 8.81)
Pleural attachment percentage	0.07 (0.05, 0.09)	0.07 (0.05, 0.09)	0.07 (0.06, 0.09)	0.07 (0.04, 0.09)	0.07 (0.06, 0.08)
Fat fraction	0.09 (0.04, 0.13)	0.06 (0.01, 0.11)	0.10 (0.05, 0.13)	0.08 (0.05, 0.14)	0.14 (0.08, 0.15)
Mean vessel density (HU)	3.37 (0.51, 15.77)	0.67 (0.12, 7.48)	4.58 (0.80, 14.82)	9.49 (0.77, 30.52)	15.57 (3.12, 40.78)
Irregularity	0.59 (0.33, 0.90)	0.42 (0.18, 0.74)	0.63 (0.39, 0.87)	0.77 (0.48, 1.00)	0.82 (0.62, 1.00)
PT (*s*)	11.10 (10.40, 12.00)	10.90 (10.50, 11.65)	11.00 (10.40, 11.88)	12.00 (10.80, 12.40)	11.55 (10.97, 12.22)
INR	1.00 (0.95, 1.06)	0.99 (0.94, 1.03)	0.99 (0.95, 1.05)	1.03 (0.97, 1.11)	1.02 (0.96, 1.09)
APTT (*s*)	28.80 (26.80, 31.20)	28.70 (26.55, 31.00)	29.10 (27.23, 31.20)	29.10 (26.80, 32.50)	28.35 (26.77, 30.47)
FIB (g/L)	3.90 (3.20, 5.11)	3.61 (2.85, 4.48)	3.83 (3.17, 5.02)	5.12 (4.05, 5.97)	3.97 (3.41, 4.99)
TT (*s*)	14.70 (13.70, 15.40)	14.80 (14.05, 15.50)	14.75 (13.62, 15.50)	14.50 (13.70, 15.20)	14.15 (13.28, 15.72)
D-D (μg/mL)	0.25 (0.11, 0.70)	0.14 (0.07, 0.55)	0.24 (0.13, 0.77)	0.39 (0.16, 0.76)	0.45 (0.21, 0.72)
CEA (ng/mL)	2.60 (1.40, 6.30)	1.40 (1.00, 2.85)	3.90 (1.70, 19.75)	3.20 (1.88, 7.65)	2.65 (1.60, 5.38)
NSE (ng/mL)	11.30 (8.30, 15.70)	9.30 (6.50, 12.95)	12.10 (9.00, 16.08)	11.95 (9.38, 16.27)	14.70 (10.30, 32.45)
CYFRA21-1 (ng/mL)	3.10 (1.90, 5.70)	2.40 (1.20, 3.45)	3.10 (1.90, 5.65)	7.35 (3.42, 17.38)	3.35 (2.15, 4.97)
SCCA (ng/mL)	0.20 (0.08, 0.44)	0.21 (0.10, 0.49)	0.18 (0.08, 0.39)	0.33 (0.18, 0.91)	0.11 (0.00, 0.21)
CA125 (IU/mL)	11.00 (6.00, 36.00)	7.00 (1.00, 11.00)	14.00 (7.00, 62.70)	14.00 (5.00, 61.25)	15.50 (8.00, 30.25)

PT: prothrombin time; INR: international normalized ratio; APTT: activated partial thromboplastin time; FIB: fibrinogen; TT: thrombin time; D-D: D-Dimer; CEA: carcinoembryonic antigen; NSE: neuron-specific enolase; CYFRA21-1: cytokeratin 19 fragment; SCCA: squamous cell carcinoma antigen; CA125: carbohydrate antigen 125; Lung-RADS: Lung Imaging Reporting and Data System.

### 2.2 *LASSO*逻辑回归分析与特征筛选结果

将*LASSO*与SMOTE一起封装到每一折的Pipeline中，对39种特征进行筛选，结果如下：二分类任务中，ResMLP模型特征筛选得到11种特征；Tab-Transformer与TT-ResMLP共得到10个特征。三分类任务中，3种模型均筛选得到13种特征，详见表2。

**表2:** 肺占位性病变良恶性诊断模型及肺癌亚型分类模型的LASSO 特征筛选结果

Features	Benign vs Malignant diagnostic model for pulmonary space-occupying lesions				Lung cancer subtype classification model		
	Tab-Transformer	ResMLP	TT-ResMLP		Tab-Transformer	ResMLP	TT-ResMLP
Gender	/	/	/		0.173	0.173	0.173
Tumor history	/	/	/		0.011	0.009	0.011
Family history	/	/	/		0.006	0.004	0.006
Ground-glass opacity	0.065	0.066	0.065		/	/	/
Part-solid nodule	/	/	/		0.003	0.003	0.003
Vessel sign	/	0.001	/		/	/	/
Calcification	/	/	/		0.021	0.020	0.021
Pleural indentation sign	0.001	0.002	0.001		/	/	/
Lung-RADS category	0.112	0.114	0.112		0.028	0.026	0.028
Age	0.029	0.027	0.029		/	/	/
Mean density	0.019	0.019	0.019		/	/	/
Surface area	/	/	/		0.118	0.116	0.118
Fat fraction	/	/	/		0.027	0.025	0.027
PT	/	/	/		0.022	0.021	0.022
FIB	0.016	0.017	0.016		0.013	0.013	0.013
TT	0.016	0.017	0.016		/	/	/
CEA	0.015	0.016	0.015		0.019	0.018	0.019
NSE	/	/	/		0.044	0.044	0.044
CYFRA21-1	0.016	0.017	0.016		/	/	/
CA125	0.011	0.013	0.011		0.020	0.019	0.020

"/" indicates that the feature was not selected by LASSO in the corresponding model.

### 2.3 SMOTE过采样结果诠释

采用SMOTE过采样对模型训练集进行样本平衡，测试集保持原始分布。以MLP分类器和t-SNE聚类的输出结果，评价SMOTE过采样的性能。结果显示：过采样后MLP分类器性能小幅提升（Precision: 0.71→0.72; Recall: 0.65→0.67; F1-score: 0.67→0.68）。t-SNE降维（困惑度=20，学习率=150）显示，SMOTE生成样本在二分类/三分类任务中与原始数据未显示严重分布偏离（图3）。

**图3:**
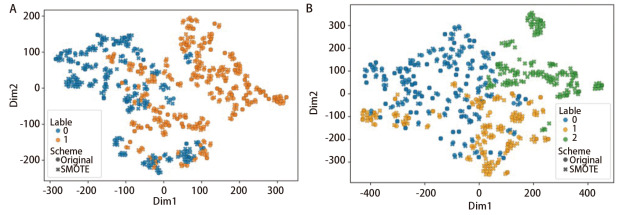
原始样本与SMOTE过采样后的t-SNE二维嵌入分布对比。A：二分类任务SMOTE前后数据分布对比图；B：三分类任务SMOTE前后数据分布对比图。横坐标为t-SNE第一嵌入维度；纵坐标为t-SNE第二嵌入维度。

### 2.4 肺占位性病变良恶性诊断及肺癌亚型分类模型构建

#### 2.4.1 肺占位性病变良恶性诊断模型训练结果

在二分类任务中，Tab-Transformer模型在测试集中表现最优，准确率及AUC值为0.78和0.86。ResMLP模型在测试集中的准确率为0.75，AUC值为0.80。TT-ResMLP模型在测试集中的AUC表现优于ResMLP模型（0.84 *vs* 0.80），但低于Tab-Transformer模型（0.84 *vs* 0.86）；测试集准确率表现优于ResMLP，但低于Tab-Transformer（0.77 *vs* 0.75 *vs* 0.78）（图4A-4C）。Tab-Transformer模型表现最优，测试集敏感性0.80、特异性0.78，具有优异的性能。

**图4:**
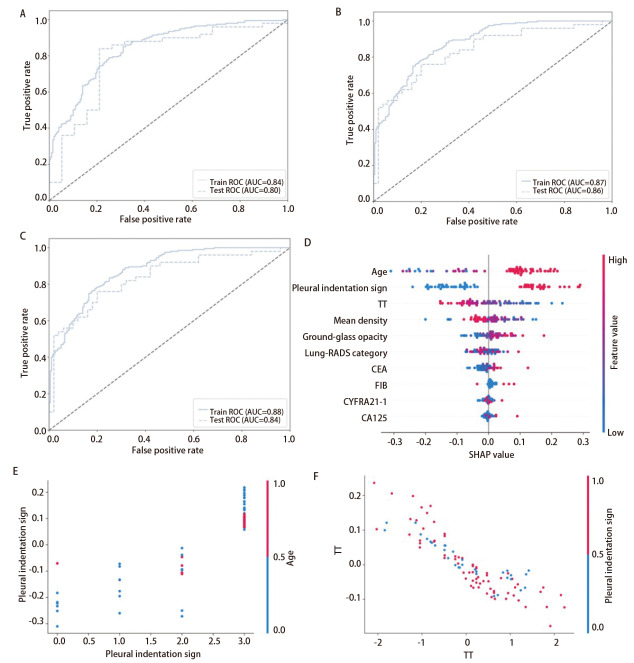
3种深度学习模型对肺占位性病变良恶性诊断模型的ROC曲线与SHAP分析。A-C：三种模型的ROC曲线（ResMLP模型、Tab-Transformer模型、TT-ResMLP模型）；D：Tab-Transformer模型的SHAP特征依赖图。点的颜色表示特征值高低（红色：高，蓝色：低），分布展示特征值与SHAP值（即对模型预测的影响）的关系；E、F：Tab-Transformer模型的特征交互可视化。横轴为特征的连续量化值（值越大特征越显著），纵轴为交互SHAP值（>0表示与协变量共同推高恶性概率，<0则共同抑制），点的颜色代表特征分层，以展示不同亚群的协同模式。

为明确各特征对肺占位性病变良恶性诊断预测结果的贡献，对表现最优的Tab-Transformer模型进行SHAP评估，特征重要性依次是：年龄、胸膜凹陷征、TT、平均密度、磨玻璃样改变、Lung-RADS分级、CEA、FIB、CYFRA21-1、糖类抗原125（carbohydrate antigen 125, CA125）（图4D）。胸膜凹陷征阳性有较高的恶性诊断贡献，且随年龄增长、TT缩短，其贡献度进一步增强（图4E、4F）。

#### 2.4.2 肺癌亚型分类模型训练结果

在多分类任务中，*LASSO*逻辑回归筛选出13种稳定特征。ResMLP模型在测试集中的准确率为0.74，AUC分别为腺癌0.89、鳞癌0.85、小细胞癌0.89，micro-ROC为0.88（图5A）；Tab-Transformer模型在测试集中的准确率为0.82，AUC分别为腺癌0.87、鳞癌0.96、小细胞癌0.93，micro-ROC为0.93（图5B）；TT-ResMLP模型在测试集中的准确率为0.87，AUC分别为腺癌0.91、鳞癌0.97、小细胞癌0.95，micro-ROC为0.95（图5C）。TT-ResMLP模型综合表现最优，该模型在测试集上表现出优异的诊断效能：腺癌（敏感性0.79、特异性0.96）、鳞癌（敏感性0.97、特异性0.94）、小细胞癌（敏感性0.91、特异性0.91），展现了模型强大的分类能力。

**图5:**
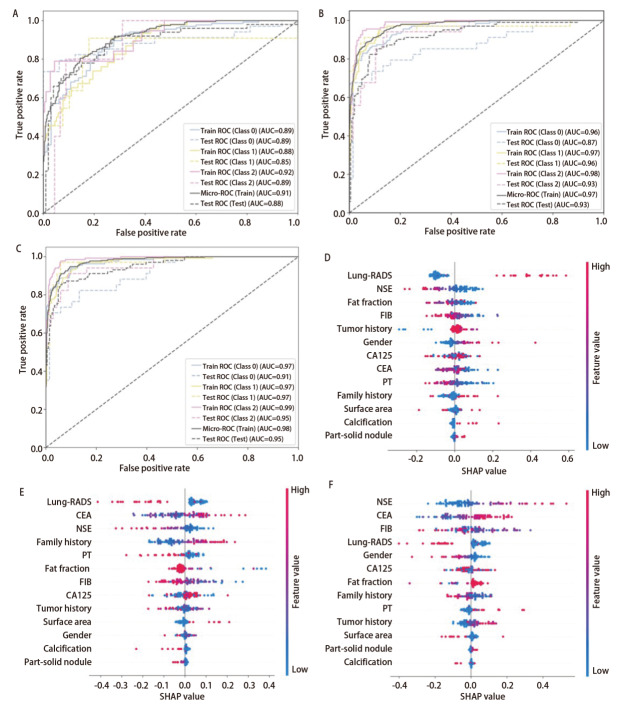
3种深度学习模型对肺癌亚型分类模型的ROC曲线及SHAP分析结果。A-C：三种模型的ROC曲线（ResMLP模型、Tab-Transformer模型、TT-ResMLP模型）：所有模型基于病理类型将患者划分为三类：Class 0（腺癌）、Class 1（鳞癌）、Class 2（小细胞癌）；D-F：TT-ResMLP模型在各亚型组别（腺癌、鳞癌、小细胞癌）中的SHAP特征贡献分析。点的颜色表示特征值高低（红色：高，蓝色：低），分布展示特征值与SHAP值（即对模型预测的影响）的关系。

为明确特征在不同亚型分组预测结果的重要性，对表现最优的TT-ResMLP模型进行SHAP分析，结果显示：腺癌分组中平均绝对SHAP值最高的5个特征为Lung-RADS分级、NSE、脂肪占比、FIB、肿瘤史；鳞癌分组中为Lung-RADS分级、CEA、NSE、家族史、PT；小细胞癌分组为NSE、CEA、FIB、Lung-RADS分级、性别（图5D-5F）。

特征交互分析结果显示：在腺癌亚组中，Lung-RADS分级升高与模型预测概率呈正相关，且PT值较低时的预测贡献更为突出。在鳞癌与小细胞癌亚组中，Lung-RADS分级升高与模型预测概率呈负相关，但男性可能增强其对鳞癌的预测贡献，NSE水平升高可提升低Lung-RADS分级对小细胞癌的预测贡献（图6A-6C）。特征决策边界分析显示：Lung-RADS分级在腺癌识别中具有较高的区分能力，而NSE在小细胞癌识别中展现出更强的区分能力（图6D）。

**图6:**
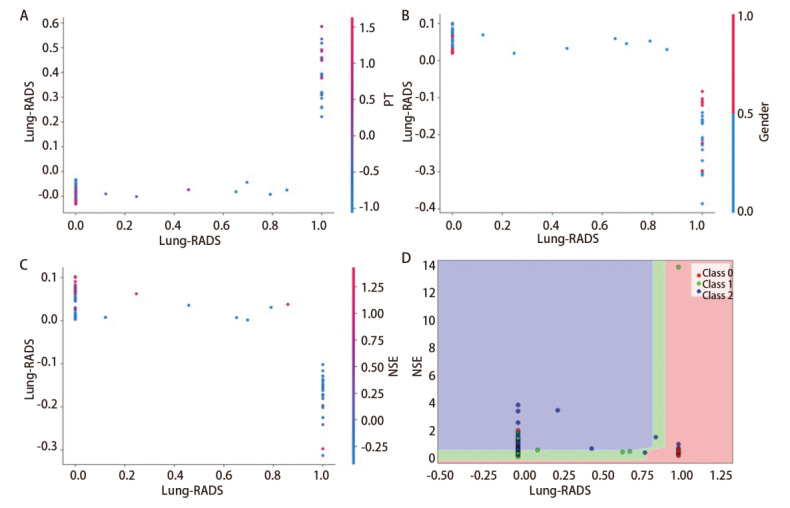
肺癌亚型分类模型特征交互图及临床决策边界分析。A：腺癌中Lung-RADS分级与PT交互图；B：鳞癌中Lung-RADS分级与性别交互图；C：小细胞癌中Lung-RADS分级与NSE交互图。横轴为特征的连续量化值（值越大特征越显著），纵轴为交互SHAP值（>0表示与协变量共同推高恶性概率，<0则共同抑制），点的颜色代表特征分层，以展示不同亚群的协同模式；D：临床决策边界分析图。横轴为经中心化处理的Lung-RADS分级（评级越高，恶性概率越大），纵轴为血清NSE浓度。图中散点按术后病理亚型着色：红色代表腺癌（Class 0），绿色代表鳞癌（Class 1），蓝色代表小细胞癌（Class 2）。

## 3 讨论

肺癌早期诊断面临传统模型泛化性弱与深度学习模型可解释性差的双重挑战。Tab-Transformer与ResMLP相结合的混合架构，兼顾了自注意力机制对特征全局依赖的捕捉能力与线性层在特征归因稳定性上的优势，其本身结构为深入理解决策过程提供了便利^[19]^。在此基础上，引入SHAP框架进行事后归因分析，可实现全局与局部的特征重要性解释，进一步提升模型决策的透明度。因此，本文通过整合多模态临床数据，构建TT-ResMLP模型并辅以SHAP可解释性分析，为提升肺占位性病变早期诊断的准确性与模型透明度提供了参考方案。

在肺占位性病变良恶性诊断任务中，Tab-Transformer综合表现最优（测试集AUC=0.86），优于ResMLP（AUC=0.80）及TT-ResMLP混合模型（AUC=0.84）。TT-ResMLP模型在测试集中的AUC表现优于ResMLP模型（0.84 *vs* 0.80），说明引入Transformer模块能够有效增强ResMLP的表征能力，弥补其在复杂特征交互建模上的不足^[17]^；但其整体性能仍略低于单一Tab-Transformer模型（0.84 *vs* 0.86），反映出混合结构在该任务中尚未完全发挥理论优势，其临床适用性尚需更大样本、多中心数据进一步验证^[22]^。在肺癌亚型分类任务中，TT-ResMLP表现最优（准确率0.87，micro-ROC 0.95），优于Tab-Transformer（准确率0.82，micro-ROC 0.93）与ResMLP（准确率0.74，micro-ROC 0.88）。这说明TT-ResMLP凭借残差连接机制有效降低了梯度消失与过拟合风险，在处理多类别分类任务时展现出更强的架构优势与临床应用潜力。

本研究基于SHAP方法解析不同特征在模型预测中的贡献。在恶性诊断中，年龄、胸膜凹陷征、磨玻璃样改变、CEA在Tab-Transformer模型中呈现正向影响，其升高/阳性与恶性风险增加相关；而TT、FIB则与恶性概率呈负相关，可能与肿瘤患者常伴高凝状态有关^[23]^。此外，胸膜凹陷征阳性对恶性的诊断贡献随年龄增长及TT缩短而增强，说明该影像学表现在特定生理与凝血环境下更能反映恶性肿瘤的生物学特性。

在肺癌亚型分类任务中，基于TT-ResMLP模型的SHAP可解释性分析揭示了不同病理亚型间显著的生物标志物差异。腺癌预测主要受益于Lung-RADS分级、肿瘤史和家族史的正向贡献，而脂肪占比、NSE、FIB和PT则呈现负向贡献。鳞癌的预测中CEA和CA125呈现正向贡献，而Lung-RADS分级、NSE、PT和FIB则起负向作用，其中，男性特征对鳞癌预测呈正向贡献，对腺癌预测则呈负向贡献，与Zhang等^[24]^报道的男性鳞癌风险较高一致。小细胞癌则呈现独特的生物标志物模式，NSE、CEA和肿瘤史对其预测呈正向作用，而Lung-RADS分级呈现负向作用。Lung-RADS分级在腺癌和小细胞癌中的差异，可能源于Lung-RADS系统主要针对表现为结节的腺癌设计，而小细胞癌多呈中央型浸润生长，影像学特征不典型，易与基于结节监测的评估体系相冲突，这反映了早期小细胞癌漏诊率较高的临床现象^[25]^。特征交互结果显示，Lung-RADS分级升高与腺癌预测概率呈正相关，且在PT值较低时其预测贡献更为显著，说明凝血功能状态可能影响该评分系统在腺癌中的鉴别能力；男性是增强Lung-RADS分级在鳞癌亚组中反向预测贡献的关键特征；NSE水平升高可提升低Lung-RADS分级对小细胞癌的预测价值。临床决策边界分析进一步发现，Lung-RADS分级在腺癌亚组中临床效用较高，而NSE效用较低；在鳞癌中两者临床效用均较低；在小细胞癌中，NSE的临床效用显著高于其他两种亚型。这些结果提示，不同病理亚型肺癌的影像学与血清标志物表现存在显著差异，在临床决策中需结合亚型特点进行综合评估。因此，未来智能诊断系统的开发需兼顾标准化分类体系与特定亚型生物学特征的协同整合，加强针对肺癌病理亚型的多模态特征挖掘^[26]^。

本研究通过*Spearman*相关性分析与*LASSO*逻辑回归筛选稳健特征，并采用SMOTE过采样技术以改善类别不平衡问题。t-SNE可视化分析显示，经SMOTE生成的样本在二分类及三分类任务中与原始数据分布基本一致，表明该方法在本研究背景下能够有效保留关键特征信息。然而也需认识到，SMOTE技术的生成效果受参数配置及特征空间结构的影响较大，在医学数据这一高维异构场景中的适应性仍有进一步提升的空间^[14]^。

综上所述，本研究通过系统比较多种深度学习架构，证实了Tab-Transformer在肺占位性病变良恶性诊断中的优势，以及TT-ResMLP混合架构在亚型分类任务中的潜力。但作为单中心回顾性研究，样本量相对有限，后续工作将重点围绕以下方向展开：（1）通过多中心合作验证模型的泛化性能；（2）开发端到端的临床应用系统，实现与现有医疗信息系统的对接；（3）开展前瞻性随机对照试验，评估模型对临床决策质量和患者预后的实际影响；（4）构建多模态数据融合诊断平台，提升小细胞肺癌等不典型表现的检出率；（5）辅助临床医生制定随访策略或手术决策。最终建立一套准确、可靠、可解释的肺癌智能辅助诊断体系，为精准医疗时代的肺癌防治工作提供有力工具。
